# 2-(4-Iodo­phen­oxy)acetamide

**DOI:** 10.1107/S1600536811025840

**Published:** 2011-07-06

**Authors:** Richard Betz, Cedric McCleland, Stephen Glover

**Affiliations:** a Nelson Mandela Metropolitan University, Summerstrand Campus, Department of Chemistry, University Way, Summerstrand, PO Box 77000, Port Elizabeth 6031, South Africa

## Abstract

The mol­ecule of the title compound, C_8_H_8_INO_2_, amide-typical resonance shortens the nominal C—N single bond to 1.322 (7) Å. In the crystal, hydrogen bonds involving both nitro­gen-bound H atoms as well as C—H⋯O contacts connect the mol­ecules into double layers approximately perpendicular to the crystallographic *b* axis. No π-stacking is apparent in the crystal structure.

## Related literature

For the crystal structure of 2-(4-nitro­phen­oxy)acetamide, see: Lakshmi Rao *et al.* (1987 ▶) and of 2-(4-chloro-2-methyl­phen­oxy)acetamide, see: Rao *et al.* (1987 ▶). For graph-set analysis of hydrogen bonds, see: Etter *et al.* (1990 ▶); Bernstein *et al.* (1995 ▶). For a description of the Cambridge Structural Database, see: Allen (2002 ▶). For the preparation, see: Glover *et al.* (1973 ▶).
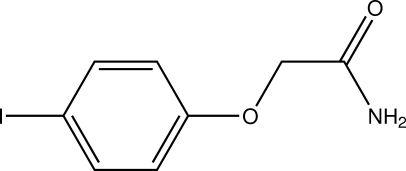


## Experimental

### 

#### Crystal data

C_8_H_8_INO_2_
*M* *_r_* = 277.05
Monoclinic, 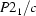

*a* = 5.1411 (4) Å
*b* = 26.473 (2) Å
*c* = 7.2960 (7) Å
β = 109.564 (3)°
*V* = 935.66 (14) Å^3^
*Z* = 4
Mo *K*α radiation
μ = 3.38 mm^−1^
*T* = 200 K
0.55 × 0.18 × 0.10 mm

#### Data collection

Bruker APEXII CCD diffractometer
Absorption correction: multi-scan (*SADABS*; Bruker, 2008 ▶) *T*_min_ = 0.824, *T*_max_ = 1.000
8332 measured reflections
2282 independent reflections
2089 reflections with *I* > 2σ(*I*)
*R*_int_ = 0.020

#### Refinement

*R*[*F* ^2^ > 2σ(*F* ^2^)] = 0.064
*wR*(*F* ^2^) = 0.120
*S* = 1.29
2282 reflections
109 parameters
H-atom parameters constrained
Δρ_max_ = 1.50 e Å^−3^
Δρ_min_ = −1.58 e Å^−3^

### 

Data collection: *APEX2* (Bruker, 2010 ▶); cell refinement: *SAINT* (Bruker, 2010 ▶); data reduction: *SAINT*; program(s) used to solve structure: *SHELXS97* (Sheldrick, 2008 ▶); program(s) used to refine structure: *SHELXL97* (Sheldrick, 2008 ▶); molecular graphics: *ORTEP-3* (Farrugia, 1997 ▶) and *Mercury* (Macrae *et al.*, 2006 ▶); software used to prepare material for publication: *SHELXL97* and *PLATON* (Spek, 2009 ▶).

## Supplementary Material

Crystal structure: contains datablock(s) I, global. DOI: 10.1107/S1600536811025840/aa2016sup1.cif

Supplementary material file. DOI: 10.1107/S1600536811025840/aa2016Isup2.cdx

Structure factors: contains datablock(s) I. DOI: 10.1107/S1600536811025840/aa2016Isup3.hkl

Supplementary material file. DOI: 10.1107/S1600536811025840/aa2016Isup4.cml

Additional supplementary materials:  crystallographic information; 3D view; checkCIF report

## Figures and Tables

**Table 1:** Hydrogen-bond geometry (Å, °)

*D*—H⋯*A*	*D*—H	H⋯*A*	*D*⋯*A*	*D*—H⋯*A*
N1—H71⋯O1^i^	0.88	2.00	2.881 (6)	178
N1—H72⋯O1^ii^	0.88	2.28	2.954 (6)	133
C2—H21⋯O1^iii^	0.99	2.48	3.422 (8)	158

Symmetry codes: (i) 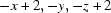; (ii) 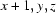; (iii) 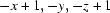.

**Fig. 1.**
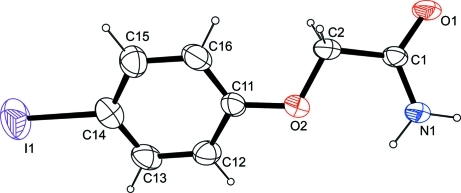
The molecular structure of the title compound, with atom labels and anisotropic displacement ellipsoids (drawn at 50% probability level).

**Fig. 2.**
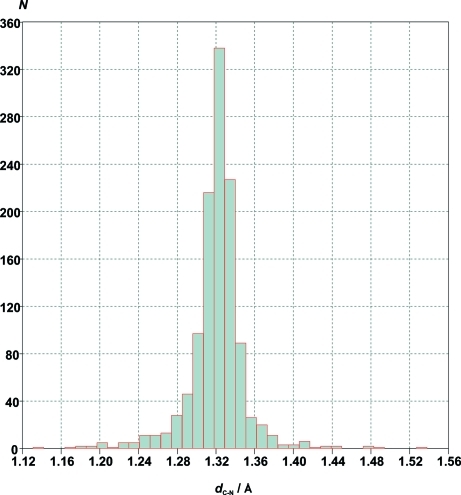
Observed distribution of C–N bond lengths in the O=C—NH2 moiety of acetamide derivatives in which the O atom is not acting as a donor atom in coordination compounds (data based on CSD search including all deposited crystal structures up to November 2010).

**Fig. 3.**
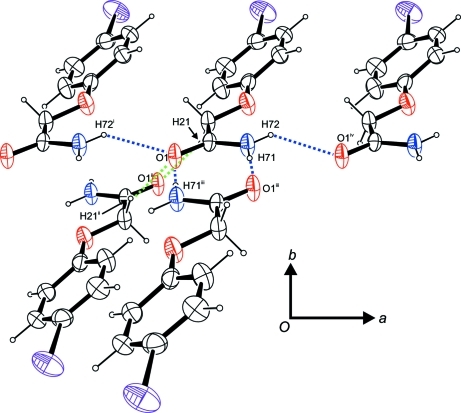
Intermolecular contacts, viewed along [0 0 - 1]. Symmetry operators: ix - 1, y, z; ii -x + 1, -y, -z + 1; iii -x + 2, -y, -z + 2; ivx + 1, y, z.

**Fig. 4.**
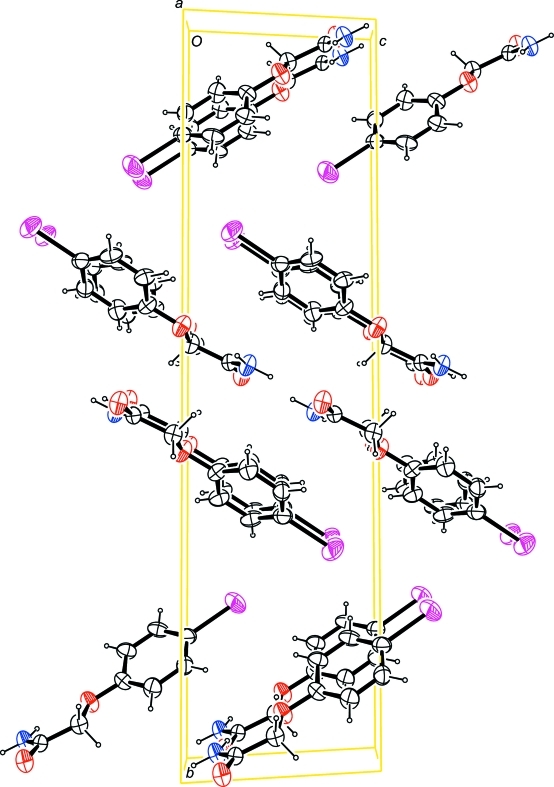
Molecular packing of the title compound, viewed along [-1 0 0] (anisotropic displacement ellipsoids drawn at 50% probability level).

## supplementary crystallographic information

### Comment

Unlike carboxylic acids, their pertaining amides have not been studied extensively as ligands in coordination chemistry. Owing to their versatility in terms of denticity, keto-enol tautomerism as well as their possible use as neutral – or upon deprotonation – anionic ligands we set out to investigate the coordination behaviour of substituted acetamide derivatives to elucidate the rules guiding the formation of complex compounds. To enable comparative studies with envisioned reaction products, we determined the molecular and crystal structure of the title compound. So far, only the structures of 2-(4-nitrophenoxy)acetamide (Lakshmi Rao *et al.*, 1987) and of 2-(4-chloro-2-methylphenoxy)acetamide (Rao *et al.*, 1987) have been discussed as examples of phenoxy-substituted derivatives of acetamide.

The C–N single bond is shortened to 1.322 (7) Å due to the amide-typical resonance. This value is in good agreement with other derivatives of acetamide whose crystallographic data has been deposited with the Cambridge Structural Database (Allen, 2002) and whose ketonic oxygen atom is not involved in donor action towards transition metals. Intracyclic C–C–C angles hardly deviate from the ideal value of 120 °. The least-squares planes defined by the acetamide moiety and the oxygen atom of the phenoxy-derivative substituent on the one hand and the carbon atoms of the carbocycle on the other hand intersect at an angle of 24.91 (29) ° (Fig. 1 and Fig. 2).

In the crystal structure, both nitrogen-bonded H atoms participate in hydrogen bonds which invariably have the carbonyl oxygen atom as acceptor. While one of the H atoms of the amino group connects the molecules to centrosymmetric dimeric subunits, the other H atom of the NH_2_ group connects these dimers to chains along [1 0 0]. Additionally, one of the hydrogen atoms of the methylene group forms a C–H···O contact whose range falls by more than 0.2 Å below the sum of van-der-Waals radii of the respective atoms. Again, the double-bonded O atom acts as acceptor. In terms of graph-set analysis (Etter *et al.*, 1990; Bernstein *et al.*, 1995), the hydrogen bonds stemming from the amino group can be described by a *C*^1^_1_(4)*R*^2^_2_(8) descriptor on the unitary level while the C–H···O contacts necessitate a *R*^2^_2_(4) descriptor on the same level. In total, the molecules are connected to double layers approximately perpendicular to the crystallographic *b*-axis (Fig. 3). No π-stacking is apparent in the crystal structure.

The packing of the compound is shown in Figure 4.

### Experimental

The compound was prepared upon reacting 2-phenoxyacetamide with *tert*-butyl hypochlorite and iodine according to a published procedure (Glover *et al.*, 1973).

### Refinement

Carbon-bound H-atoms were placed in calculated positions (C—H 0.99 Å for the methylene group and C—H 0.95 Å for aromatic carbon atoms) and were included in the refinement in the riding model approximation, with *U*(H) set to 1.2*U*_eq_(C). Nitrogen-bound H-atoms were placed in calculated positions (N—H 0.88 Å) and were included in the refinement in the riding model approximation, with *U*(H) set to 1.2*U*_eq_(N).

### Crystal data

**Table:**

C_8_H_8_INO_2_	*F*(000) = 528
*M**_r_* = 277.05	*D*_x_ = 1.967 Mg m^−^^3^
Monoclinic, *P*2_1_/*c*	Mo *K*α radiation, λ = 0.71073 Å
Hall symbol: -P 2ybc	Cell parameters from 5293 reflections
*a* = 5.1411 (4) Å	θ = 3.1–28.2°
*b* = 26.473 (2) Å	µ = 3.38 mm^−^^1^
*c* = 7.2960 (7) Å	*T* = 200 K
β = 109.564 (3)°	Rod, colourless
*V* = 935.66 (14) Å^3^	0.55 × 0.18 × 0.10 mm
*Z* = 4	

### Data collection

**Table:**

Bruker APEXII CCD diffractometer	2282 independent reflections
Radiation source: fine-focus sealed tube	2089 reflections with *I* > 2σ(*I*)
graphite	*R*_int_ = 0.020
φ and ω scans	θ_max_ = 28.2°, θ_min_ = 3.1°
Absorption correction: multi-scan (*SADABS*; Bruker, 2008)	*h* = −6→6
*T*_min_ = 0.824, *T*_max_ = 1.000	*k* = −34→35
8332 measured reflections	*l* = −8→9

### Refinement

**Table:**

Refinement on *F*^2^	Primary atom site location: structure-invariant direct methods
Least-squares matrix: full	Secondary atom site location: difference Fourier map
*R*[*F*^2^ > 2σ(*F*^2^)] = 0.064	Hydrogen site location: inferred from neighbouring sites
*wR*(*F*^2^) = 0.120	H-atom parameters constrained
*S* = 1.29	*w* = 1/[σ^2^(*F*_o_^2^) + (0.0042*P*)^2^ + 6.0795*P*] where *P* = (*F*_o_^2^ + 2*F*_c_^2^)/3
2282 reflections	(Δ/σ)_max_ < 0.001
109 parameters	Δρ_max_ = 1.50 e Å^−^^3^
0 restraints	Δρ_min_ = −1.58 e Å^−^^3^

### Fractional atomic coordinates and isotropic or equivalent isotropic displacement parameters (Å^2^)

**Table:**

	*x*	*y*	*z*	*U*_iso_*/*U*_eq_	
I1	0.81735 (14)	0.20849 (2)	−0.25506 (9)	0.0685 (2)	
O1	0.6872 (8)	0.01765 (17)	0.7947 (6)	0.0390 (10)	
O2	1.0024 (8)	0.08086 (17)	0.4969 (6)	0.0416 (10)	
N1	1.1340 (9)	0.0300 (2)	0.8286 (7)	0.0367 (11)	
H71	1.1917	0.0152	0.9432	0.044*	
H72	1.2543	0.0422	0.7781	0.044*	
C1	0.8667 (10)	0.0339 (2)	0.7322 (8)	0.0278 (11)	
C2	0.7756 (11)	0.0587 (2)	0.5354 (9)	0.0359 (13)	
H21	0.6883	0.0332	0.4343	0.043*	
H22	0.6365	0.0850	0.5302	0.043*	
C11	0.9411 (12)	0.1079 (2)	0.3245 (9)	0.0345 (12)	
C12	1.1409 (13)	0.1428 (2)	0.3187 (10)	0.0399 (14)	
H12	1.3013	0.1474	0.4296	0.048*	
C13	1.1073 (13)	0.1706 (2)	0.1538 (11)	0.0425 (15)	
H13	1.2466	0.1936	0.1489	0.051*	
C14	0.8721 (14)	0.1650 (2)	−0.0038 (10)	0.0408 (14)	
C15	0.6678 (15)	0.1314 (3)	0.0021 (10)	0.0440 (15)	
H15	0.5036	0.1281	−0.1069	0.053*	
C16	0.7036 (13)	0.1029 (3)	0.1658 (10)	0.0435 (15)	
H16	0.5647	0.0797	0.1699	0.052*	

### Atomic displacement parameters (Å^2^)

**Table:**

	*U*^11^	*U*^22^	*U*^33^	*U*^12^	*U*^13^	*U*^23^
I1	0.0910 (5)	0.0588 (3)	0.0581 (3)	−0.0160 (3)	0.0282 (3)	0.0173 (3)
O1	0.0201 (19)	0.062 (3)	0.038 (2)	−0.0006 (18)	0.0138 (17)	0.007 (2)
O2	0.027 (2)	0.056 (3)	0.042 (2)	−0.0059 (19)	0.0109 (18)	0.014 (2)
N1	0.022 (2)	0.055 (3)	0.035 (3)	0.001 (2)	0.012 (2)	0.011 (2)
C1	0.022 (3)	0.035 (3)	0.029 (3)	0.001 (2)	0.013 (2)	−0.001 (2)
C2	0.022 (3)	0.049 (3)	0.038 (3)	−0.002 (2)	0.012 (2)	0.003 (3)
C11	0.030 (3)	0.037 (3)	0.041 (3)	0.007 (2)	0.018 (3)	0.010 (2)
C12	0.031 (3)	0.035 (3)	0.051 (4)	−0.002 (2)	0.012 (3)	0.005 (3)
C13	0.039 (3)	0.031 (3)	0.063 (4)	−0.004 (3)	0.024 (3)	0.008 (3)
C14	0.052 (4)	0.032 (3)	0.046 (4)	0.001 (3)	0.026 (3)	0.000 (3)
C15	0.050 (4)	0.047 (4)	0.035 (3)	−0.007 (3)	0.014 (3)	−0.001 (3)
C16	0.038 (3)	0.054 (4)	0.044 (4)	−0.010 (3)	0.020 (3)	0.000 (3)

### Geometric parameters (Å, °)

**Table:**

I1—C14	2.102 (6)	C11—C16	1.380 (9)
O1—C1	1.236 (6)	C11—C12	1.392 (8)
O2—C11	1.389 (7)	C12—C13	1.371 (9)
O2—C2	1.415 (6)	C12—H12	0.9500
N1—C1	1.322 (7)	C13—C14	1.370 (10)
N1—H71	0.8800	C13—H13	0.9500
N1—H72	0.8800	C14—C15	1.388 (9)
C1—C2	1.503 (8)	C15—C16	1.372 (9)
C2—H21	0.9900	C15—H15	0.9500
C2—H22	0.9900	C16—H16	0.9500
			
C11—O2—C2	116.2 (4)	C13—C12—C11	120.3 (6)
C1—N1—H71	120.0	C13—C12—H12	119.8
C1—N1—H72	120.0	C11—C12—H12	119.8
H71—N1—H72	120.0	C14—C13—C12	119.7 (6)
O1—C1—N1	123.2 (5)	C14—C13—H13	120.1
O1—C1—C2	118.2 (5)	C12—C13—H13	120.1
N1—C1—C2	118.6 (5)	C13—C14—C15	120.5 (6)
O2—C2—C1	110.9 (4)	C13—C14—I1	119.7 (5)
O2—C2—H21	109.5	C15—C14—I1	119.8 (5)
C1—C2—H21	109.5	C16—C15—C14	119.8 (6)
O2—C2—H22	109.5	C16—C15—H15	120.1
C1—C2—H22	109.5	C14—C15—H15	120.1
H21—C2—H22	108.0	C15—C16—C11	120.0 (6)
C16—C11—O2	125.4 (5)	C15—C16—H16	120.0
C16—C11—C12	119.6 (6)	C11—C16—H16	120.0
O2—C11—C12	115.1 (5)		
			
C11—O2—C2—C1	−175.7 (5)	C12—C13—C14—C15	−0.1 (10)
O1—C1—C2—O2	172.4 (5)	C12—C13—C14—I1	179.0 (5)
N1—C1—C2—O2	−8.5 (8)	C13—C14—C15—C16	−1.1 (10)
C2—O2—C11—C16	−20.4 (9)	I1—C14—C15—C16	179.8 (5)
C2—O2—C11—C12	159.1 (5)	C14—C15—C16—C11	0.5 (10)
C16—C11—C12—C13	−2.6 (9)	O2—C11—C16—C15	−179.2 (6)
O2—C11—C12—C13	177.9 (6)	C12—C11—C16—C15	1.3 (10)
C11—C12—C13—C14	2.0 (10)		

### Hydrogen-bond geometry (Å, °)

**Table:**

*D*—H···*A*	*D*—H	H···*A*	*D*···*A*	*D*—H···*A*
N1—H71···O1^i^	0.88	2.00	2.881 (6)	178
N1—H72···O1^ii^	0.88	2.28	2.954 (6)	133
C2—H21···O1^iii^	0.99	2.48	3.422 (8)	158

Symmetry codes: (i) −*x*+2, −*y*, −*z*+2; (ii) *x*+1, *y*, *z*; (iii) −*x*+1, −*y*, −*z*+1.
